# Alterations in Gene Array Patterns in Dendritic Cells from Aged Humans

**DOI:** 10.1371/journal.pone.0106471

**Published:** 2014-09-05

**Authors:** Jia-ning Cao, Anshu Agrawal, Edward Sharman, Zhenyu Jia, Sudhir Gupta

**Affiliations:** 1 Division of Basic and Clinical Immunology, Department of Medicine, University of California Irvine, Irvine, California, United States of America; 2 Department of Neurology, University of California Irvine, Irvine, California, United States of America; 3 Department of Statistics, University of Akron, Akron, Ohio, United States of America; 4 Department of Family and Community Medicine, Northeast Ohio Medical University, Rootstown, Ohio, United States of America; 5 Pathology & Laboratory Medicine, University of California Irvine, Irvine, California, United States of America; University of California Riverside, United States of America

## Abstract

Dendritic cells (DCs) are major antigen-presenting cells that play a key role in initiating and regulating innate and adaptive immune responses. DCs are critical mediators of tolerance and immunity. The functional properties of DCs decline with age. The purpose of this study was to define the age-associated molecular changes in DCs by gene array analysis using Affymatrix GeneChips. The expression levels of a total of 260 genes (1.8%) were significantly different (144 down-regulated and 116 upregulated) in monocyte-derived DCs (MoDCs) from aged compared to young human donors. Of the 260 differentially expressed genes, 24% were down-regulated by more than 3-fold, suggesting that a large reduction in expression occurred for a notable number of genes in the aged. Our results suggest that the genes involved in immune response to pathogens, cell migration and T cell priming display significant age-related changes. Furthermore, downregulated genes involved in cell cycle arrest and DNA replication may play a critical role in aging-associated genetic instability. These changes in gene expression provide molecular based evidence for age-associated functional abnormalities in human DCs that may be responsible for the defects in adaptive immunity observed in the elderly.

## Introduction

Aging is characterized by a progressive decline in immune responses, resulting in an increased susceptibility to infections and impaired response to vaccination [1]–[2]. Paradoxically, aging is associated with chronic inflammatory state [3]. and an increased incidence of diseases associated with inflammation including atherosclerosis, Alzheimer's disease, and arthritis [4]. The molecular mechanisms responsible for this paradox are not well characterized.

Dendritic cells (DCs) play a critical role in bridging innate and adapotive immune response [5]–[6]. Upon antigen capture, immature DCs become activated and migrate to the lymphoid organs where they acquire co-stimulatory molecules, toll-like receptors (TLRs) and chemokine receptors, and prime lymphocytes to initiate an adaptive immune response. In aged humans, several functions of DCs are compromised including phagocytosis, uptake of antigens, and migration [5]. There is an aberrant cytokine secretion by various DC subsets, including increased basal levels of pro-inflammatory cytokines [7]–[8], but their response to foreign antigens upon stimulation is decreased [7], [9]. In contrast, the immunogenicity to self-antigens as a result of epigenetic changes is increased [10]–[11], suggesting a loss of peripheral self-tolerance. Interferon type I and type III secretion by plasmacytoid dendritic cells and monocyte-derived DCs (MoDCs) and the capacity of DCs to prime naïve T cell subsets are also impaired in aged humans [12]–[13].

In this investigation, we have compared differential gene expression patterns in MoDCs from young and aged subjects using a fold-change cutoff of ≥1.5. We have (1) identified the gene expression patterns from aged MoDCs, (2) demonstrated that expression changes associated with aging occur in genes involved in immune response, cell cycle and response to oxidative stress, and (3) provided candidate genes for further studies. These age-associated changes in gene expression patterns in MoDCs provide molecular bases for age-associated functional abnormalities in immune responses, which may play an important role in the progressive decline in adaptive response in aging.

## Materials and Methods

### Blood donors

Blood was collected from healthy aged and young donors. Young donors ranged from 20–30 years of age and elderly donors were between 75–90 years of age. Description of cohort used for microarray analysis is provided in Table 1 while Table 2 provides description of donors for PCR and cell cycle analysis. Aged healthy subjects are of middle class social state and living independently. Each donor was requested to discontinue any vitamins, minerals and antioxidants one week prior to blood draw. This study was approved by the Institutional Review Board of the University of California, Irvine. All participants signed their written informed consent forms.

**Table 1:** MoDCs donor characteristics for microarray data.

Donor Array ID	Age	Sex
Young 1	22	Female
Young 2	25	Female
Young 3	27	Female
Young 4	20	Male
Aged 1	84	Female
Aged 2	77	Female
Aged 3	78	Female
Aged 4	87	Female
Aged 5	81	Male

**Table 2:** MoDCs donor characteristics for qRT-PCR and cell cycle.

Donor qRT-PCR ID	Age	Sex
Y1-p*	28	Male
Y2-p*	26	Male
Y3-p*	32	Female
Y4-p*	23	Female
Y5-p*	25	Female
Y6-p	22	Male
Y7-p	24	Male
Y8-p	27	Female
Y9-p	25	Female
Y10-p	29	Female
A277-p*	76	Male
A164-p*	94	Female
A268-p*	95	Female
A146-p*	85	Male
A276-p*	85	Female
A163-p	87	Female
A103-p	81	Male
A244-p	84	Female
A83-p	72	Male
A276-p	85	Female

*Cell cycle analysis of DNA content in DCs from these aged and young donors.

### Preparation of human monocyte-derived dendritic cells

MoDCs were prepared essentially as described [7], [14]. Briefly, monocytes were purified from the PBMCs by positive selection using CD14 magnetic beads (Stemcell Sep, Vancouver, BC). The purity of the isolated CD14^+^ monocytes was >90% as determined by flow cytometry (FACS). To induce differentiation of DCs, purified CD14^+^ monocytes were cultured in RPMI 1640 10% fetal bovine serum. 50 ng/ml supplemented with human rGM-CSF and 10 ng/ml human rIL-4 (Peprotech, Rocky Hill, NJ). Half of the medium was replaced every 2 days and MoDCs were collected after 6 days. The purity of the MoDCs was >95% as determined by the expression of CD14^−^ CD11c+ and HLA-DR+.

### RNA extraction and purification

Total RNA was extracted from MoDCs of aged and young subjects using the TRI Reagent kit (Molecular Research Center Inc., Cincinnati, OH, USA), following the manufacturer's protocol. The integrity of intact total RNA was verified with an Agilent 2100 Bioanalyzer (Agilent Technologies, Palo Alto, CA, USA).

### Real-time quantitative reverse transcriptase PCR

qRT-PCR was performed in MoDCs from ten young and ten aged donors (Table 2, Donor qRT-PCR). 1 µg of total RNA was reverse transcribed to cDNA by using the High Capacity cDNA Reverse Transcription Kit (Applied Biosystems) with random hexamers as primers following the manufacturer's instructions. The cDNA was amplified by PCR in a 20-µL reaction mixture containing 1 µL of 20 µM primers, 2 µL of SYBR Green PCR Master Mix (Applied Biosystems) and 4 µL of cDNA (50x diluted from original synthesized cDNA) or water as a negative control.

Initial denaturation of DNA was carried out at 95°C for 10 min; forty amplification cycles were performed, each cycle consisting of denaturation (95°C, 30 s) and annealing with extension (65°C, 1 min). Each sample was amplified in triplicate, and results were normalized with GAPDH gene expression as ‘housekeeper.’ A 4-log absolute standard curve (synthesized by Ziren Research LLC, Irvine, CA) [15] dilution series was run using each primer pair at optimal concentration, and amplification efficiencies were calculated. The fold changes of differential expression between healthy young donors and aged subjects were calculated using the ratios of each gene of interest to GAPDH. For a list of primer sequences, see Table S3.

### Gene array processing and statistical analysis

MoDC samples from the four young and five aged donors (Table 1) were examined for gene expression profile by a method previously described [16] using 9 HG-U133A_2 Gene Array chips (Affymetrix, Santa Clara, CA, USA, http://www.affymetrix.com). “All microarray procedures were performed at the Gene Array Technology Facility, University of California, Irvine. Each chip contains 22,277 25-mer probe sets corresponding to 18450 unique transcripts and 14500 genes. A 10 µg aliquot of total RNA from each sample was processed and applied to a HG-U133A_2 Gene Array chip according to the manufacturer's protocol (http://www.affymetrix.com/support/technical/manual/expression_ manual.affx). Microarray Command Console V3.1 (Affymetrix) was used for gene expression image analysis and quality control. Affymetrix default settings were used, and statistical parameters such as background, noise, and spike-in controls were found to be within acceptable limits. Ratios of 3′ to 5′ mRNA transcripts of constitutively expressed internal housekeeping gene controls, human β-actin and glyceraldehyde-3-phosphate dehydrogenase (GAPDH), were also measured to be within the recommended Affymetrix guidelines. Expression Console Software (Affymetrix) was used to produce CEL files. Web-based Genetic Analysis Software (www. Genesifter.net, Geospiza, Seattle, WA, USA) was used to analyze the data. By uploading each CEL file to Genesifter's website, normalized gene expression levels were obtained using GC-RMA. Initial analysis focused on removing genes that did not meet our criteria for differential expression by applying t-tests followed by the Benjamini-Hochberg method for false discovery rate (FDR) adjustment” [16]–[17]. “Genes were accepted for further analysis if they changed by more than 1.5 fold and had an adjusted p≤0.05 (by t-test) with an FDR <5%. Lists of genes with expression changes meeting our criteria were filtered by the Gene Ontology (GO) program by performing biological function analyses. Degree of enrichment for biological processes and molecular functions of interest were assessed by analyzing z-scores generated by the GO program or by applying a *p* value <0.05. The z-score statistically rates a GO category by evaluating whether the number of genes included in the category is either greater or less than that expected by chance [18], using as criteria either z-scores >2.0 (genes are over-represented in the corresponding GO category) or <−2.0 (under-represented). Hierarchical clustering analysis [19] was performed using the program CLUSTER from Genesifter” [16]. Microarray data measured in HG-U133A_2 Gene Array chips of mRNA samples of DCs from 4 young and 5 aged donors are submitted to GEO, accession number: GSE 58015.

### Cell cycle analysis of DNA content by FACS in DCs from aged and young donors

Cell cycle analysis of DNA content was performed in MoDCs from five young and five aged donors (Table 2, **Donor qRT-PCR**). MoDCs about (5×10^5^) were washed with Phosphate Buffered Saline (PBS) and incubated in ice-cold 70% ethanol/30% PBS at 4°C for 1 h. Cells were then washed once with PBS, resuspended in 0.5 ml PBS plus 0.5 ml DNA extraction buffer (0.2 M Na_2_HPO_4_, 0.4 µM citric acid, pH 7.8), incubated for 5 min at room temperature. Finally, cells were resuspended in 1 ml of staining solution [20 µg/ml RNase A (Qiagen), 20 µg/ml propidium iodide (Sigma) in PBS], and incubated for 1 h in the dark at room temperature. Ten thousand cells were acquired on FACS Calibur and analysis was done using the FlowJo cell cycle program.

## Results

### Global characteristics of age-associated gene expression changes in human MoDCs

**Figure 1:**
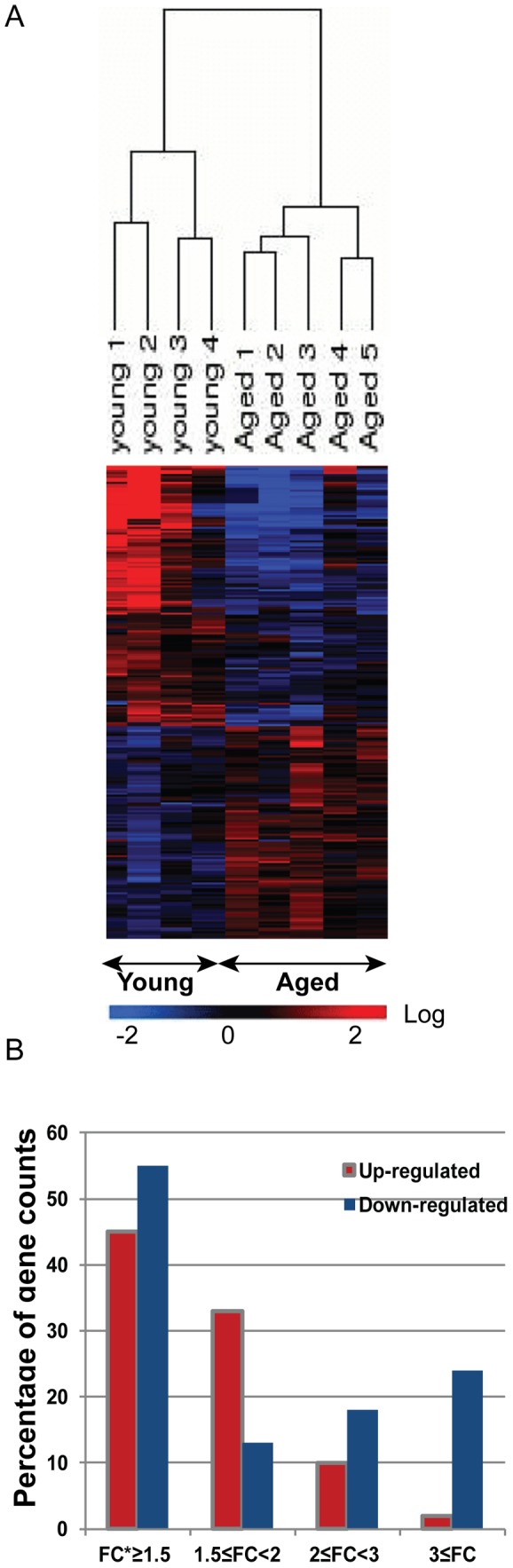
Clustering of Gene expression changes in young and aged MoDCs. (A) Hierarchical clustering heat map of 260 genes. with significant expression changes in young and aged MoDCs. Each column represents an individual donor; each row refers to a gene. Gene expression changes with respect to median changes across age are denoted by: red, up-regulated (ratio ≥1.5); blue, down-regulated (ratio <1/1.5); and black, unchanged. (B) Distribution of 260 genes by expression size, as percentages of total numbers of up- or down-regulated genes. *:FC  =  Fold change.

In this study, we analyzed the differential expression of genes in MoDCs from four young and five aged (Table 1) healthy human donors using Affymetrix GeneChip HG U133A 2.0 microarrays. Of the 14,500 well-characterized genes measured, 260 genes (1.8%) displayed significant donor age-associated expression changes. Although the total numbers of up-regulated and down-regulated genes were approximately similar (1.24∶1.0 down:up ratio), 24% of the down-regulated genes displayed differential changes more than three-fold, indicating that the levels of some genes were highly reduced in MoDCs from aged donors as compared to young subjects (Fig. 1A, B, Table S1).

**Figure 2:**
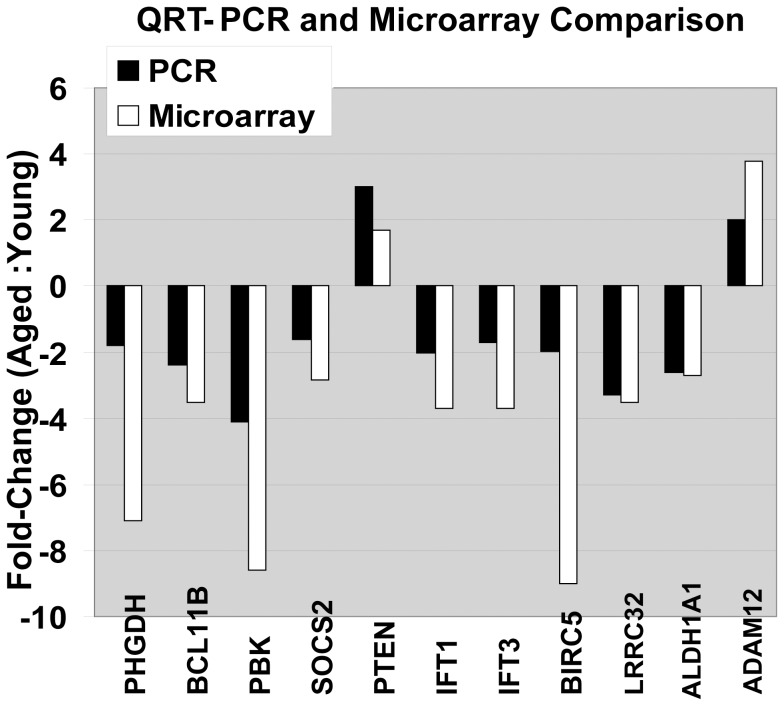
Validation of microarray data with real-time quantitative RT-PCR. qRT-PCR validation. Black bars represent microarray hybridizations, while white bars represent values from qRT-PCR. Ratio of expression for each gene (aged group to young group) is shown as fold change. The Pearson correlation coefficient between the qRT-PCR and microarray data was 0.64.

In addition, we carried out real-time quantitative RT-PCR (qRT-PCR) analysis in triplicate for 10 selected genes using cDNA from MoDCs from ten young and ten aged individuals. (Table 1B). The age-related changes in gene expression measured by qRT-PCR were in agreement with microarray data for those specific genes (Fig. 2). The Pearson correlation coefficient between the qRT-PCR and microarray data was 0.64, (Fig. 2; for gene ID and primer sequences see Table S3).

### Functional association of expression changes in MoDCs from aged donors

**Figure 3:**
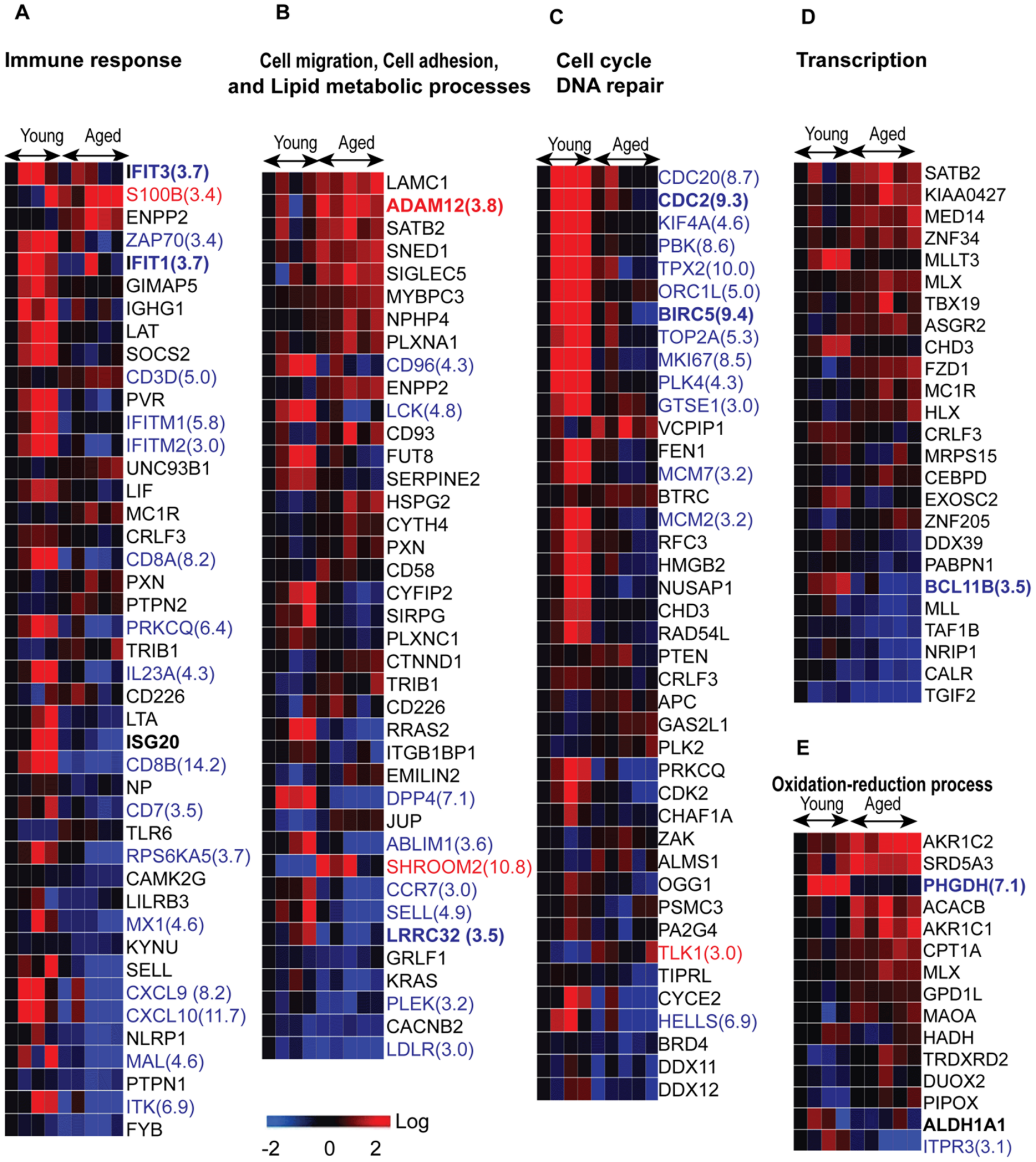
Heat maps of expression changes of genes in cell processes affected by donor age. (A–F) Heat maps of the genes by functional group (for detailed gene information, see Table S2, worksheets A, B). Expression of genes in red text were increased, and of genes in blue decreased, by ≥3 fold. Each column represents a donor; each row refers to a gene.

Age-associated expression of 209 genes filtered by Gene Ontology and involved in specific cellular processes were either suppressed or induced. Expression levels of many of these genes were highly reduced (>3 fold), with down-regulated genes being over-represented in several categories including *immune response*, *cell migration* and *cell cycle* (Fig. 3A–C, Table 3; Table S2). Genes with altered expression involved in *immune response* included: *interferon-stimulated* genes *ISG20, MX1, CXCL10*, and *CXCL9*; IFN-induced transmembrane proteins *IFITM1* and *IFITM2*; and IFN-induced proteins with tetratricopeptide repeats *IFIT1* and *IFIT3*. Similarly modulated genes involved in *DC cell migration* and *regulation of T cell activation* included *IL23, CCR7*, *and DDP4*; those involved in the *cell surface receptor-linked signaling pathway* included *CD8A, CD8B*, *SIRPG, ITK* and *LCK*; and finally genes involved in the *toll-like receptor signaling pathway* included *RPS6KA5, TLR6* and *Unc93B1*. The downregulated genes involved in *cell cycle* and DNA repair included *CDC20*, *CDC2, ORC1* (*ORC1L*), *BIRC5, MCM7, MCM2, CDK2, PBK, TPX2, ZAK, PSMC3*, *ZAK*, *APC, FEN1, RFC3, OGG1, CHD3, and DSCC1*. Other downregulated *cell cycle* genes, such as *TPX2*, *BIRC5*, *KIF4A*, and *GTSE1* play key roles in spindle assembly and are required for mitosis and for response to DNA damage repair. That many genes are highly down-regulated (≥3-fold) may indicate that aged-donor DCs are less likely to respond to an immune challenge and that the integrity of these cells may be impaired.

**Table 3:** Numbers of genes (total in list, up- and down-regulated) and associated z-scores >|2.0| in selected functional categories that contained significantly over-represented differentially expressed genes in naïve aged-donor DCs compared to young.

Ontology	List	Up	Down	z-up	z-down
cellular process	209	91	118	**2.28**	**2.88**
**I. signaling transduction**	93	41	52	1.22	**2.57**
1. response to stress	61	18	42	−0.34	**4.1**
2. cell surface receptor linked signaling pathway	54	18	36	0.34	**3.84**
3. immune system process	57	15	42	0.15	**7.08**
4. immune response	46	8	38	−0.26	**9.52**
5. defense response	34	8	26	−0.22	**9.01**
6. locomotion	37	14	23	1.6	**3.48**
7. cell migration	22	10	12	**2.28**	**2.0**
8. chemotaxis	19	7	10	0.35	**2.42**
9. antigen receptor-mediated signaling pathway	8	0	8	−0.84	**8.63**
**II. localization**	93	56	37	**3.25**	1.38
1.transport	68	39	29	**2.22**	−0.47
2. cellular localization	41	20	21	**2.1**	1.53
3. protein localization	31	15	16	**2.08**	1.1
4. transmembrane transport	24	16	8	**3.69**	0.32
5. cell adhesion	23	16	7	**2.42**	0.9
6. cell morphogenesis	25	15	6	**3.66**	0.93
**III. cell cycle**	42	7	35	−1.44	**5.28**
1. cell cycle arrest	18	1	17	−1.6	**4.1**
2. DNA replication	10	0	10	−1.7	**3.72**
**IV. oxidation-reduction process**	16	13	3	**2.09**	−1.6

In contrast, expression levels of genes involved in the processes of o*xidation-reduction* and *cell adhesion* were modestly increased (Fig. 3B, E, Table 3; Table S2). The genes involved in *oxidation-reduction* included *AKR1C2, AKR1C1, SRD5A3, ACACB, CPT1A, MAOA, DUOX2*; and those involved in *cell adhesion* include *HSPG2, JUP, CD93, LAMC1, CTNND1, PXN, SNED1, CYTH4* and *SIGLEC5*. These results indicate that aged DCs may be in a state of chronic oxidative stress. In addition, *PHGDH*, (a gene encoding a metabolic enzyme that plays a critical role in serine biosynthesis in the phosphorylation pathway) [20] was downregulated (Fig 3E).

### More cells were arrested in G1 phase in MoDCs from aged donors as compared to MoDCs from young donors

**Figure 4:**
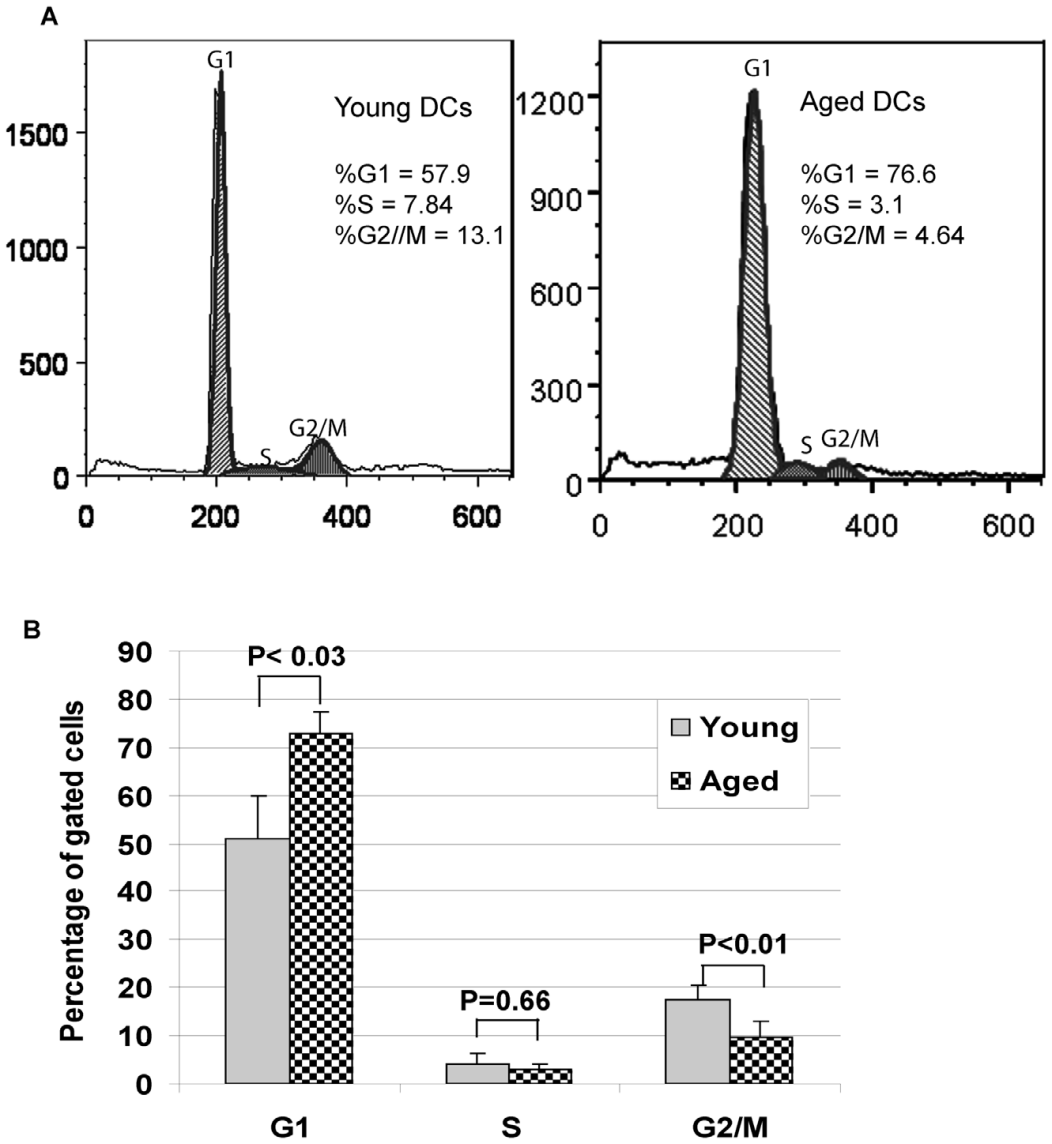
Cell cycle phases are different between MoDCs from aged and young donors. (A) Distribution of aged and young MoDCs existing in the various phases of the cell cycle as determined by FACS. (B) Statistical analysis and percentages of MoDCs from young and aged donors in each phase of the cell cycle (n = 5, each group). Donors' information sees Table 2.

Cell cycle analysis of DNA content in DCs from aged and young donors demonstrated significantly increased number of cells in G1 phase (73% *vs* 51%, p<0.03) from old donors compared to young donors. In contrast, fewer cells were in G2/M phase (9.8% *vs* 17.4%, p<0.01). These results indicate that more cells were arrested in G1 phase in aged DCs than in young (Fig. 4), a profile reminiscent of senescent cells.

## Discussion

Aging is characterized by a progressive decline in the adaptive immune response and chronic inflammation. The functionality of dendritic cells, both for orchestrating the immune response and for maintaining immune tolerance, is compromised with age [5]. Identifying the functions and signaling pathways of genes that exhibit substantial (≥1.5-fold) expression changes in MoDCs from healthy aged human donors provides insights into the molecular mechanisms underlying immunosenescence.

Expression levels of genes belonging to several critical functional groups, most notably immune defense response against viruses, were reduced in MoDCs from aged donors. Type I Interferons play a critical role in defense against viral infections as they not only interfere with viral replication but also lead to further activation of DCs, macrophages via the induction of set of genes known as interferon-stimulated genes. Our data demonstrates substantial down-regulation (by about 3-fold) in the expression of several interferon-stimulated genes, such as *IFITM1*, *IFITM2*, *IFIT1, IFIT3, ISG20*, and *MX* (Fig. 3A, Table S2), in MoDCs from aged subjects. IFIT and IFITM each denote a genetically and functionally distinct family of IFN-stimulated genes that have antiviral activities against a range of human viruses [21]–[22]. IFIT1 binds to the free 5′-ppp moiety on RNA of a number of viruses, including influenza A virus, and inhibits infection by forming a complex with IFIT2 and IFIT3 that sequesters viral nucleic acid [22]. Therefore, decreased IFIT1 and IFIT3 gene expression and decreased expression of IFITM1 and IFITM2 suggest that in aging there is an impairment of both IFN-I production and IFN-induced signaling mechanisms. ISG20 is an interferon-inducible 3′-5′ exonuclease that inhibits replication of several human and animal RNA viruses [23]–[24]. Mx1 (Myxoma resistance protein 1 or human MXA) is a member of a family of large GTPases that are induced exclusively by IFN-I and have broad antiviral activity against several viruses, including influenza A [25]. We have previously reported that both plasmacytoid dendritic cells and MoDCs from aged donors secrete reduced levels of type I interferons in response to influenza virus [12]. Our present data suggests that aged DCs are compromised not only with respect to the production of type I interferon but also with regard to the signaling response to IFN-I that contribute to the increased susceptibility of aged subjects to viral infections.

DCs sense and capture pathogens via the expression of pathogen recognition receptors (PRRs), such as Toll-like receptors (TLR), which are critical for the development of adaptive immunity [26]–[27]. Our data show age-related alterations in several critical genes in the TLR signaling pathway (Fig 3A, Table S2), including upregulation of the gene encoding Unc93B1; this factor interacts with TLRs -3, -7, and -9 and is involved in the production of pro-inflammatory cytokines [28]. In contrast, expression of the *SOCS2* gene [29], which acts as a negative regulator of TLR-induced DC activation, was down-regulated. Defects in negative-feedback loops regulating inflammation lead to increased inflammation-sensitive autoimmune diseases [30]. Thus, the alterations in Unc93B1 and SOCS2 may result in an enhancement of TLR activation-associated inflammation [31]–[32]. This is consistent with our previous studies where we have reported an increase in inflammatory cytokine, TNF-a and IL-6 secretion from MoDCs of elderly in response to Lipopolysaccharide [7] More recently, we have also demonstrated that MoDCs from elderly are deficient in the production of anti-inflammatory cytokine, IL-10 which controls inflammation [12]. The T cells primed by these inflammatory DCs also display reduced IL-10 production. Furthermore, the DCs from elderly were also found to be defective in maintaining tolerance in that they displayed enhanced basal level of activation and increased reactivity to self-antigens [10]. Several genes which are involved in the tolerizing function of DCs appear downregulated in the elderly [Figure 3]. These include AldhA1 which codes for RALDH enzymes which plays an important role in maintenance of mucosal tolerance by DCs [33] (Fig. 2, 3). A reduction in the expression of KYNU is also observed in the DCs from aged (Figure 3). KYNU encodes of Kynureninase and is involved in tryptophan metabolism which is one of the pathways in DCs involved in the generation of T regulatory cells [34]. Furthermore, we have observed reduced expression of LRRC33 which is also important for generation of regulatory T cells [35] (Fig. 2,3). Aged DCs also express increased levels of metalloproteinases such as ADAM12 (Fig. 2, 3). We have reported that this enhances the permeability of airway epithelial cells barrier leading to increased airway inflammation [36].

Dendritic cells (DCs) are critical for adaptive immunity and tolerance, and the migration and accurate positioning of DCs is indispensable for the priming of T cells and immune surveillance. Several genes involved in these processes were downregulated (Fig. 3A, B, and Table S2). *CCR7*, with an approximately 3-fold reduction in expression, was prominent; its product is a receptor required for the migration of DCs and T cells to the areas of lymph nodes where T cell priming occurs [37]–[38]. The protein encoded by *ITGB1BP1* (integrin beta 1 binding protein 1), which binds to small GTPase Cdc42, also plays an important role in controlling cell adhesion and migration [39], and antigen-uptake by DCs [40]. Cdc42 by itself is also a major regulator of endocytosis in DCs [41]. Our functional studies support these hypotheses as we have previously reported impairment in DC antigen uptake and migration [7] in the aged subjects. *Dipeptidylpeptidase 4* (*DPP4/CD26*), co-localizes with membrane-bound adenosine deaminase on human DCs and enhances the ability of the latter to stimulate T-cell proliferation [42]. *CD58* (*lymphocyte function-associated antigen 3*, *LFA-3*), is significant in that its molecular interaction with CD2 counter-receptors mediates the ability of monocytes to augment T cell activation [24]. Protein products of the genes *ITK*, *LCK*, and *SIPG* are also involved in T cell-mediated immunity. Of these, ITK is a member of the family of Tec kinases, and is known to have an important role in CD4+ T cell differentiation [43]. Therefore, the reduced expression of *ITK* in aged DCs may explain decreased priming ability and T cell proliferation in aged humans. Therefore, downregulation of DPP4, CD58 and ITK would be consistent with our observation of impaired capacity of aged MoDCs to prime T cells [11].

In addition to immune defense, our data show that the expression levels of many genes involved in *chromatin organization*, *cell cycle arrest*, *DNA replication* and *repair* and *regulation of transcription* were reduced by more than 3-fold in aged-donor cells (Fig. 3C, Table S2). Genes involved in *cell cycle arrest* included *CDC20*, *CDC2, ORC1* (*ORC1L*), *BIRC5, MCM7, MCM2, CDK2, PBK, TPX2, ZAK*; those involved in *DNA replication* and *repair* included *PSMC3, HELLS, MLL, FEN1, RFC3, OGG1, CHD3*, and *DSCC1*. Cellular degeneration, uncontrolled cell proliferation and genomic instability are major hallmarks of aging [44]–[45]. CDC2, a cyclin-dependent kinase that normally drives cells into mitosis, is also the ultimate target of pathways that mediate rapid arrest in G2 in response to DNA damage [46]. CCNE2 (CYCE2) is a CDK2 partner in the late G1 and S phases of the mammalian cell cycle [47]. ORC1L (*ORC1*), a component of ORC (origin of replication complex), is required for efficient cell-cycle progression (transition from G1 to S phase) and DNA replication [48]. Some of these genes encode microtubule-associated proteins such as *TPX2* [24], [49]. *BIRC5* [50] and *GTSE1* play a key role in spindle assembly and are required for mitosis and for responding to DNA damage. GTSE1 is also required for cell migration and for microtubule-dependent disassembly of focal adhesions [51]. KIF4A is associated with chromosomes during mitosis and mediates the interaction between mitotic chromatin and microtubules [52]. FEN1 is a structure-specific nuclease with 5′-flap endonuclease and 5′-3′ exonuclease activities and is involved in DNA replication and repair [53]. The *RFC3* gene encodes DNA replication factor C subunit 3, a protein that maintains genome stability by inhibiting cell cycle progression in the presence of DNA damage or incomplete replication [54]. *HELLS* encodes a helicase involved in DNA repair, while the protein encoded by *MLL* mediates modifications to chromatin associated with epigenetic transcriptional activation [55]–[56].*CHD3* encodes chromodomain helicase DNA binding protein 3, a member of a family of chromatin remodeling proteins involved in repression of gene expression [57]. We have previously demonstrated that type I and type III interferon promoter genes display increased association with the repressor histone, H3K9me3 in the unstimulated state in MoDCs from the elderly while their association with activator histone, H3K4me3 is reduced on activation with influenza virus [12].

In contrast to DNA repair genes, three genes important in cell cycle regulation, *PTEN*, *ZAK* and *APC*, were elevated. PTEN expression has both a potent inhibitory effect on DNA synthesis and the ability to block cell cycle progression in G1 [58]. Overexpression of PTEN results in decreased PKB/Akt phosphorylation and cyclin E/cdk2 activity, and inhibits S-phase entry in MCF-7 cells [59]. We have also observed an increased expression of PTEN at both mRNA and protein levels, and a reduced degree of AKT phosphorylation in aged MoDCs [7]. A greater percentage of cells from aged donors were observed to be in G1, along with a lower percentage of these cells in M phase (Fig. 4). Notably, cellular senescence is a state of stable cell cycle arrest [60] that occurs not only in normally proliferating cells, but also in arrested quiescent or terminally differentiated cells [61]. The significant changes that were observed in cell cycle-associated genes, despite the fact that DCs are terminally differentiated, suggests that these genes may be playing a very important role in the maintenance of cellular architecture, chromatin structure, cell cycle arrest and DNA repair, all of which are fundamentally important for the maintenance of DC cell integrity, genomic stability and basic functions.

Genes involved in *transcription regulation* that were downregulated in aged-donor MoDCs included *CALR*, *TAF1B, PABPN1*, *CEBPD* and *Bcl11b* (Fig 3C, D). Calreticulin (*CALR*) acts as an important modulator of the regulation of gene transcription mediated by nuclear hormone receptors [62]. *TAF1B* and *PABPN1* are essential for the initiation of transcription by RNA polymerase I. Investigating the roles these genes in the age-related changes in DC cellular integrity, may provide new insights into the age-associated functional decline of DCs.

The present study utilizes monocyte derived DCs which display both similarities and differences than DCs found in circulation or tissues. Both circulating and in vitro–differentiated MoDCs share the main functional properties of APCs by priming naive T cells and inducing immune tolerance. They also display similar phenotypic expression of DC markers such as low expression of CD14, CD40, CD80 and high expression of CD11c and MHCII [63] Even though both DCs are of myeloid lineage. MoDCs are more homogenous population compared to peripheral DCs, which are divided into two major DC populations expressing CD1c and CD14. Genome-wide expression profiling has demonstrated that MoDC are more similar to inflammatory DCs which are DC derived from monocytes in an inflammatory setting than circulatory DCs. Nevertheless, studies of both are equally relevant as inflammatory DCs have been demonstrated to play a major role in protection against lung infection of influenza virus [64]. The study of MoDCs thus provides insight into the age-associated changes in the functions of monocytes, inflammatory DCs and some shared functions of circulatory DCs.

By comparing expression changes associated with donor age, we observed that such changes in DCs were greater than those observed in aged CD8 + T cells [16] (Fig. S1). A substantially larger number of genes, that are involved in immune responses and cellular integrity, were down-regulated over 3-fold (24% vs. 4%) in MoDCs. Therefore, despite the fact that donor age altered fewer genes in MoDCs as compared to aged CD8+ T cells, the greater magnitude in expression of downregulated genes nevertheless may result in significant alterations in MoDC functions.

In summary, by utilizing microarray gene analysis of MoDCs from young and aged donors, we have identified distinctive age-related gene expression patterns in MoDCs from aged donors. Genes with expression changes associated with donor age appear to play important roles in fundamental cellular processes such as immune response, cellular and DNA integrity, and oxidation-reduction. These changes may alter the functionality of aged human MoDCs, and impair adaptive immunity and exacerbate inflammation in the elderly.

## Supporting Information

Figure S1 **Percentages of up-and down-regulated genes from aged-donor DCs compared to aged-donor CD8+ T cells.**
(TIF)

Table S1 **A list of 260 genes with expression changes in DCs associated with donor age and meeting the criteria of** ***p*** **<0.05 (student's** ***t*** **-test), FDR <5%, and fold change ≥1.5.**
(XLS)

Table S2 **Classification by cellular function of genes displayed in** **Figure 3** **.** These are genes with age-associated expression changes in cells derived from 4 young and 5 aged individual donors. Expression level means, standard errors, p-statistics and per-array values are tabulated.
(XLS)

Table S3 **Sequences of primers used for qRT-PCR.**
(XLS)
