# Supplementary figures and images for: Alterations in Gene Array Patterns in Dendritic Cells from Aged Humans
Source: PLoS One. 2014 Sep 5;9(9):e106471. doi: 10.1371/journal.pone.0106471 (PMC4156347; doi:10.1371/journal.pone.0106471)

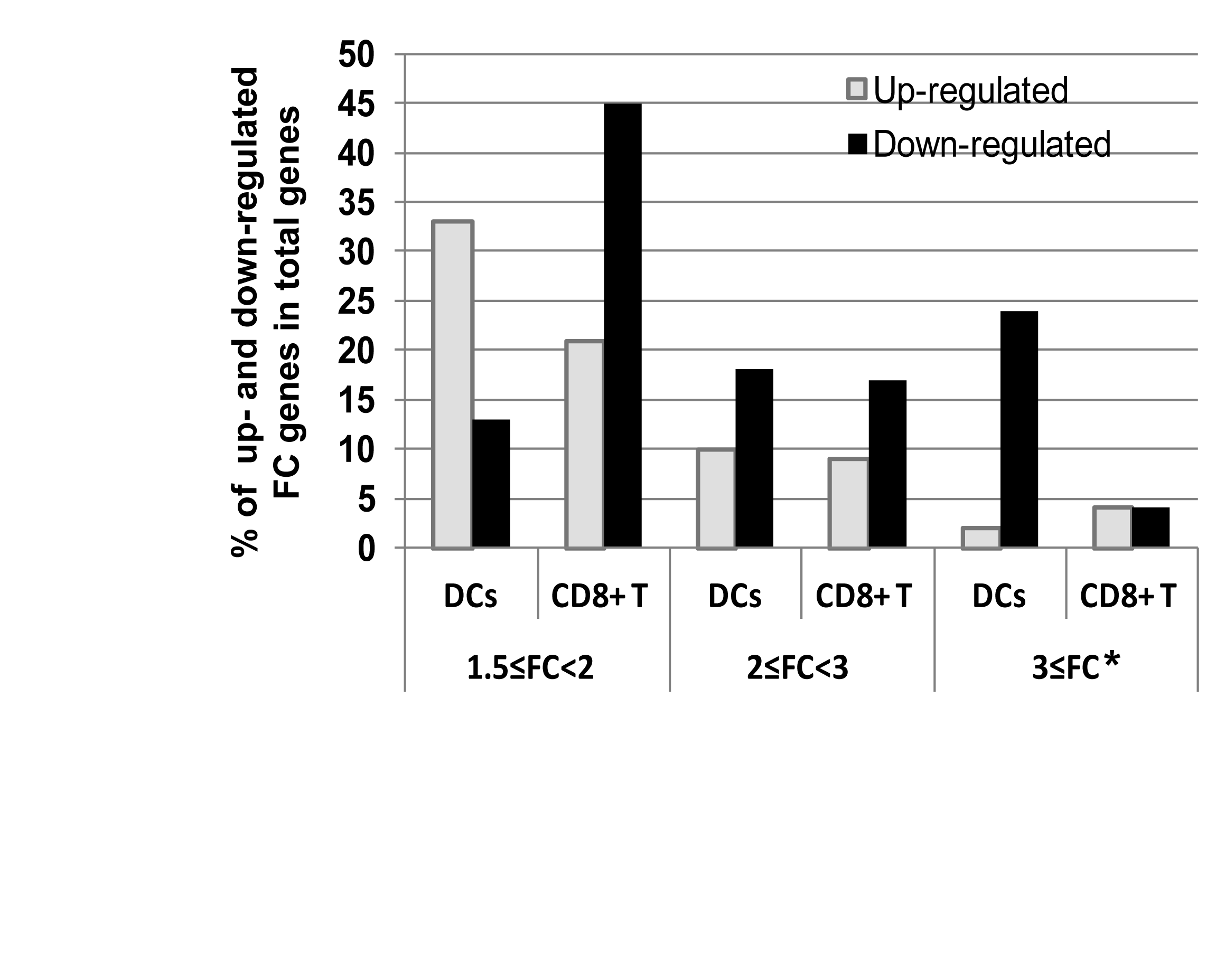

Supplement: Figure S1 — Percentages of up-and down-regulated genes from aged-donor DCs compared to aged-donor CD8+ T cells. (TIF) [file pone.0106471.s001.tif]
